# *Aeromonas veronii*-associated endogenous endophthalmitis: a case report

**DOI:** 10.1186/s13256-024-04412-7

**Published:** 2024-03-20

**Authors:** Jiali Lin, Haibin Zhong, Qi Chen, Ling Cui, Fan Xu, Fen Tang

**Affiliations:** Department of Ophthalmology, The People’s Hospital of Guangxi Zhuang Autonomous Region & Guangxi Key Laboratory of Eye Health & Guangxi Health Commission Key Laboratory of Ophthalmology and Related Systemic Diseases Artificial Intelligence Screening Technology, Nanning, 534000 Guangxi China

**Keywords:** *Aeromonas veronii*, Endophthalmitis, Case report

## Abstract

**Background:**

*Aeromonas veronii* is a very rare and highly pathogenic microorganism. We investigate the clinical characteristics and significance of endogenous endophthalmitis caused by *Aeromonas veronii* in our patient.

**Case presentation:**

A 30-year-old Asian women with systemic lupus erythematosus, uremia, and hypertension developed acute infectious endophthalmitis caused by *Aeromonas veronii*. After emergency vitrectomy and antibiotic therapy, the clinical condition worsened requiring enucleation.

**Conclusions:**

*Aeromonas veronii* can cause infection in the human eye, which can manifest as acute endophthalmitis. Early diagnosis and targeted therapy are important for successful treatment.

## Introduction

*Aeromonas veronii* is an anaerobic gram-negative bacillus that is widespread in the aquatic environment, which was originally defined by Hickman-Brenner et al. (1987) as a new species that can cause gastroenteritis, urinary tract infections, biliary tract infections, and necrotizing fasciitis [1–3]. Although the number of reported cases of *Aeromonas veronii* infection has gradually increased in recent years, endogenous endophthalmitis (EE) caused by *Aeromonas veronii* is still a rare finding. Here, we present a case of acute infectious EE caused by infection with *Aeromonas veronii* in an immunocompromised state.

## Case presentation

**Fig. 1:**
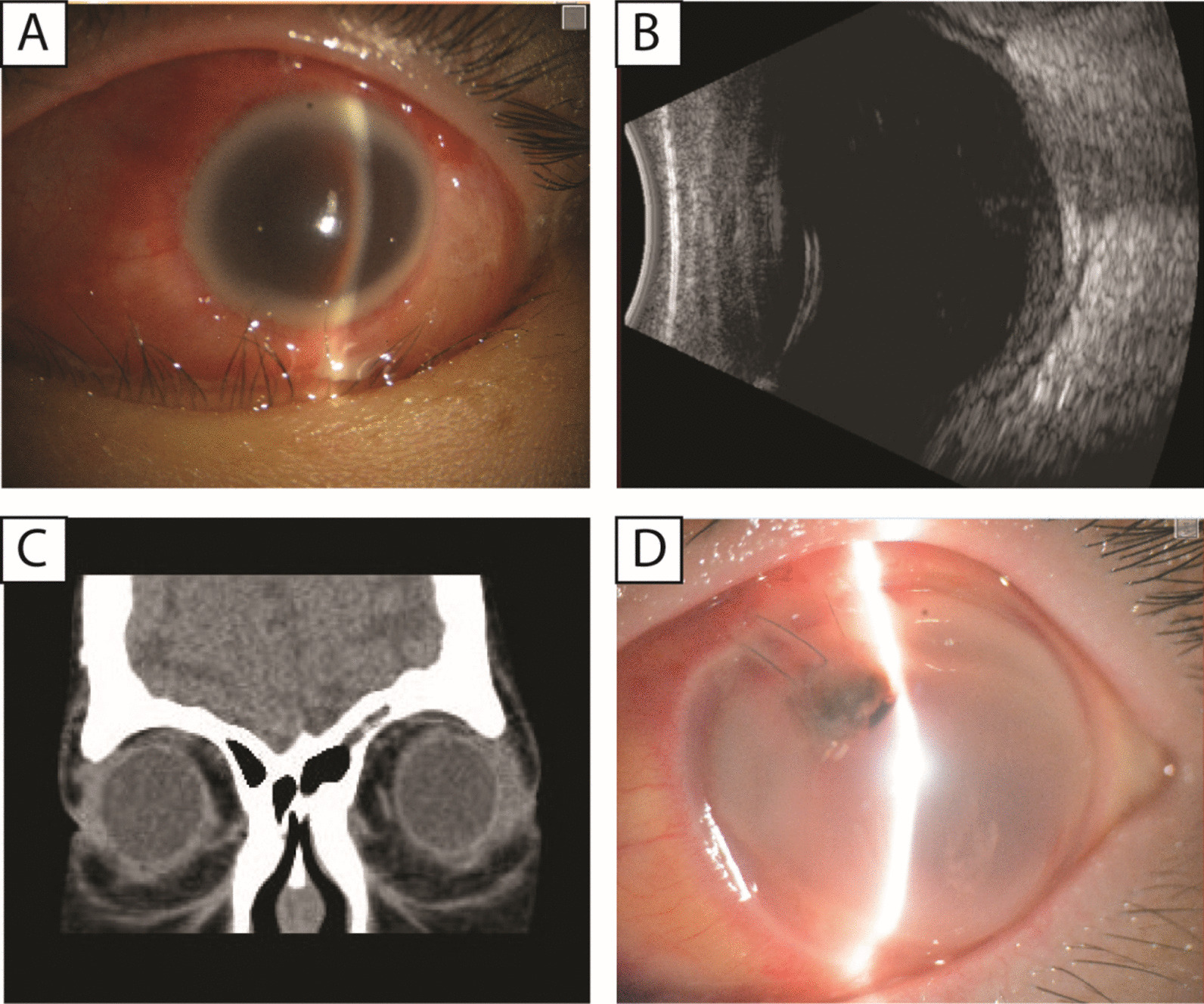
**A** Slit-lamp examination revealed severe conjunctival hyperemia, corneal edema, and hypopyon. **B** Ultrasound B scans showed dotted, clustered low to moderate echogenicity in the posterior vitreous and edema and thickening of the bulbous wall. **C** Computed tomography scan showed periocular tissue swelling. **D** Severe corneal ulceration and perforation

A 30-year-old Asian female patient presented to our emergency department with sudden loss of vision accompanied by redness and pain for 1 day, with no history of trauma or ocular disease. She had been taking prednisone acetate (10 mg/d) for 8 years for systemic lupus erythematosus (SLE). The patient had end-stage renal disease and was on hemodialysis (HD). Comprehensive ophthalmic examinations were performed. Best-corrected visual acuity was 20/32 in the right eye, and no light perception in the left eye. Intraocular pressure (IOP) was 13.6 mm Hg in the right eye and undetectable in the left eye, as measured by non-contact tonometry. There was upper lid erythema and edema, marked proptosis with no motility in the left eye. As shown in Fig. 1A, slit-lamp examination revealed severe conjunctival hyperemia, corneal edema, and hypopyon. Fundus visualization was poor due to the corneal opacity, edema, and vitreous opacities. Ultrasound B-scans showed dotted, clustered low to moderate echogenicity in the posterior vitreous and edema and thickening of the bulbous wall as shown in Fig. 1B. Computed tomography (CT) scan showed periocular tissue swelling as shown in Fig. 1C.

A systemic workup for the source of infection was performed. Laboratory testing showed that white blood cells were at normal levels (4.39 × 10^9^/L), CRP was elevated at 30.37 mg/L. Kidney and liver function were severely impaired (serum creatinine 551 µmol/L, aspartate aminotransferase 241 U/L). There was no presence of liver abscesses, sinus infections, endocarditis, meningitis, or indwelling catheters detected by X-ray, CT, or abdominal ultrasound. In addition, the patient was afebrile and the blood culture was negative. Overall, no source of infection was found.

This patient had no history of trauma or surgery, and was in an immunocompromised state. Endogenous endophthalmitis with extra-orbital involvement was diagnosed. Pars plana vitrectomy with silicone oil implant was performed subsequently. Vitreous humor sample was collected for diagnosis and microbial identification. In addition, antibiotics were administered systematically (ceftazidime 2 g IV BID) and locally (levofloxacin eye drop QID). to control infection prior to microbiological culture and antibiotic susceptibility testing (AST) results. Microbiological culture which based on the Matrix-Assisted Laser Desorption/Ionization-Time-of-Flight (MALDI-TOF) showed that a rare bacterium- *Aeromonas veronii* was isolated from the vitreous fluid. In addition, the AST showed that this type of bacteria was sensitive to second-generation cephalosporins and quinolones, including both ceftazidime and levofloxacin. However, endogenous endophthalmitis progressed rapidly, leading to severe corneal ulceration and perforation on post-operative day 1 as shown in Fig. 1D. Enucleation was performed on postoperative day 2 after vitrectomy because of protrusion of intraocular contents and no possibility of visual recovery. Neutrophilic infiltration and abscess formation were suggested by pathological examination of the ocular contents. Two weeks after the surgery, there was no signs of recurrent infection.

## Discussion

We reported a case of EE caused by *Aeromonas veronii*, the patient is a young woman was diagnosed with SLE, and was in an immunocompromised state due to prolonged use of corticosteroids and immunosuppressive drugs. After aggressive antibiotic treatment and vitrectomy, the condition continued to deteriorate and the eye was eventually enucleated. *Aeromonas veronii* was identified in the vitreous fluid.

*Aeromonas* is a genus of gram-negative, facultative anaerobic, rod-shaped bacteria. Infection in humans is relatively rare, from the few reports available, the *Aeromonas* mainly cause gastroenteritis, cholecystitis, respiratory infections, urinary tract infections, peritonitis, etc. [4–6]. The four major *Aeromonas* species are *Aeromonas caviae* (37.26%), *Aeromonas dhakensis* (23.49%), *Aeromonas veronii* (21.54%), and *Aeromonas hydrophila* (13.07%) [6]. By analyzing our case and previous studies [7–9], Fatimah Alibrahim have reported an example of *Aeromonas veronii* infection that progressed from cellulitis of the thighs to bilateral eye globe rupture in a very short period of time, and finally died of septic shock, it appears that infection with Aeromonas veronii has more severe symptoms, rapid progression, and is more destructive. Following the guidelines of Infectious Diseases Society of America (IDSOA), in our case, we administered both a second-generation cephalosporin (ceftazidime) and quinolones (levofloxacin) based on the AST results, which was also consistent with the recommendation of IDSOA. After timely treatment with the combination of surgery and antibiotic therapy, the inflammation in the periorbital tissues was greatly reduced. However, the eye was still deteriorating rapidly and the prognosis was poor.

In our case, a careful and thorough examination of the patient to locate the primary focus of infection did not reveal any skin sores, inflammation of the dialysis tubing, or other signs of infection. Previous studies have shown that *Aeromonas veronii* is widely distributed in the aquatic environment [10]. Therefore, we hypothesized that the source of the infection in this patient was caused by the ingestion of contaminated food or water, which entered the bloodstream through the gastrointestinal tract and then crossed the blood-ocular barrier into the vitreous, causing the disease. Although this patient's blood cultures were negative, previous studies have demonstrated that even for other common sources of infection, blood cultures are only 50% positive, let alone for this rare bacterium.

Endophthalmitis is one of the most devastating eye infections that can be classified as endogenous or exogenous, EE is much less common than exogenous endophthalmitis, accounting for only 2–8%. EE can be attributed 50% to bacteria and the remaining 50% to fungi, is extremely difficult to diagnose and treat, with clinical symptoms typically including vision loss, eyelid edema, redness, photophobia, and pain. A retrospective analysis of endogenous endophthalmitis found that predisposing conditions were present in 60% of patients, most commonly in patients with diabetes, malignancy and immunocompromised [11]. Usually, EE was associated with liver abscess, endocarditis and urinary tract infection [12], in our patients, there was no evidence of these infections, however, we have found the presence of echogenicity in the vitreous cavity using ocular ultrasound, and previous reports have suggested that significant vitreous cavity involvement is the hallmark of EE [13]. Early diagnosis and appropriate treatment are key factors in preserving vision. Treatment of EE includes the use of systemic and ocular antibiotics, and early pars plana vitrectomy (PPV) is beneficial for severe intraocular infections [14]. However, due to the extreme destructive power of *Aeromonas veronii*, early PPV was unable to save the patient's vision in our case.

SLE is a chronic autoimmune connective tissue disease common in Asian women that can affect multiple organs, with ocular involvement in up to one-third of patients, most commonly presenting as dry keratoconjunctivitis [15, 16]. The pathology of ocular involvement by autoimmunity in SLE has been extensively studied [17, 18], but the side effects of treatment for SLE have not been widely reported. In the treatment regimen of SLE, aggressive immunosuppression has been effective in improving the prognosis of SLE, but the occurrence of treatment-related side effects also seriously affects the long-term quality of life and prognosis of patients. In one statistic, infection was found to be the second leading cause of death in SLE patients [18]. In our case, prolonged use of prednisone acetate leads to a decrease in the body's immune function and increases the risk of opportunistic infections [19].

In conclusion, we report a case of endogenous endophthalmitis caused by *Aeromonas veronii* that occurred in an immunocompromised patient with an unknown source of infection and resulted in rapid deterioration despite prompt diagnosis and treatment.

## Conclusions

This case should remind clinicians of the destructive power of bacteria, especially in immunocompromised patients with an unknown source of infection and rapid disease progression, early detection is critical and thorough eye examinations are essential to save vision.

## Data Availability

Data sharing is not applicable to this article as no datasets were generated or analysed during the current study.

## Abbreviations

SLE: Systemic lupus erythematosus
EE: Endogenous endophthalmitis
AST: Antibiotic susceptibility testing
IDSOA: Infectious Diseases Society of America
PPV: Pars plana vitrectomy
